# Preparation and testing of cefquinome-loaded poly lactic-co-glycolic acid microspheres for lung targeting

**DOI:** 10.1080/10717544.2017.1321058

**Published:** 2017-04-28

**Authors:** Shaoqi Qu, Li Zhao, Jiajia Zhu, Chunmei Wang, Cunchun Dai, Hui Guo, Zhihui Hao

**Affiliations:** 1 Agricultural Bio-Pharmaceutical Laboratory, Qingdao Agricultural University, Qingdao, China and; 2 National-Local Joint Engineering Laboratory of Agricultural Bio-Pharmaceutical Technology, Qingdao, China

**Keywords:** Cefquinome, poly lactic-co-glycolic acid microspheres, target, sustained release, *in vivo*

## Abstract

The aim of this study was to prepare cefquinome-loaded poly lactic-co-glycolic acid (PLGA) microspheres and to evaluate their *in vitro* and *in vivo* characteristics. Microspheres were prepared using a spry drier and were characterized in terms of morphology, size, drug-loading coefficient, encapsulation ratio and *in vitro* release. The prepared microspheres were spherical with smooth surfaces and uniform size (12.4 ± 1.2 μm). The encapsulation efficiency and drug loading of cefquinome was 91.6 ± 2.6 and 18.3 ± 1.3%, respectively. *In vitro* release of cefquinome from the microspheres was sustained for 36 h. *In vivo* studies identified the lung as the target tissue and the region of maximum cefquinome release. A partial lung inflammation was observed but disappeared spontaneously as the microspheres were removed through *in vivo* decay. The sustained cefquinome release from the microspheres revealed its applicability as a drug delivery system that minimized exposure to healthy tissues while increasing the accumulation of therapeutic drug at the target site. These results indicated that the spray-drying method of loading cefquinome into PLGA microspheres is a straightforward method for lung targeting in animals.

## Introduction

Cefquinome sulfate (CEQ) is a fourth-generation cephalosporin used to treat animals due to its broad antimicrobial activity against both Gram-positive and Gram-negative bacteria (Michael et al., 1991). It is used for treatment of acute mastitis and foot rot in cattle and respiratory tract diseases in pigs, cows, and horses (Shpigel et al., 1997). CEQ is active against *Escherichia coli, Serratia marcescens, Bacteroides fragilis, Clostridium prazmowski, Erysipelothrix rhusiopathiae* as well as species of *Bacillus, Klebsiella, Streptococcus, Pasteurella, Salmonella* and *Actinobacillus in vitro* (Chin et al., 1992).

The efficacy of CEQ is frequently limited by its inability to reach the target site especially when CEQ is administered in conventional drug dosage forms or delivery systems (Vasseur et al., 2014). Targeted drug delivery increases the drug quantity at the target site while simultaneously decreasing the systemic load to other body regions. Alternative dosage forms that provide this capacity include liposomes and nanoparticles (Fu et al., 2013; Kefeng et al., 2015). Lung-targeted drug delivery systems can deliver drug to the lung *via* inhalation or intravenous administration (Villara et al., 2016). However, drug targeting to the lung periphery *via* inhalation can be problematic due to the presence of inflammation or mucus plugs (Hassanpour Aghdam & Ghanbarzadeh et al., 2016). Furthermore, nanoparticle inhalation can result in epithelial cell toxicity so intravenous administration of lung-targeted drugs is a more viable delivery option (Luo et al., 2015).

Microspheres have sustained release formulations that maintain a targeted concentration of drug over a specific period (Ahsan et al., 2002). This drug delivery system has emerged as a remedial measure to improve site-specific drug delivery and to improve therapeutic responses and reduce adverse effects (Zhang et al., 2011). Drugs implanted in microspheres are absorbed by the capillaries at the injection site and enter systemic circulation *via* lymphatics. Their distribution to the target organ is then governed by pharmacodynamics, which can bypass the first pass effect and avoid pre-systemic elimination in the liver encountered with oral administration (Wang et al., 2016).

Poly lactic-co-glycolic acid (PLGA) are a group of biocompatible and biodegradable polymers that are well defined for *in vivo* use (Anderson & Shive, 2012). The rapid degradation rate of low molecular weight PLGA (lactide:glycolide 50:50) complies well with biodegradability requirements for polymers destined for pulmonary delivery, and can be extensively degraded within 1 week (Diab et al., 2012). In the current study, we developed PLGA-loaded microspheres for intravenous administration of CEQ in order to improve its lung targeting ability. We prepared polymeric microspheres by spray drying and characterized their surface morphology and size distribution. We evaluated drug-loading coefficients, encapsulation ratios and *in vitro* release properties and investigated tissue distribution.

## Materials and methods

### Materials

PLGA (molecular weight = 20 000; PLA/PGA = 75/25), sodium phosphate dibasic dehydrate (Na_2_HPO_4_·2H_2_O), sodium phosphate monobasic (NaH_2_PO_4_) were purchased from Sigma-Aldrich (St. Louis, MO). CEQ (2.5%) was obtained from MSD Animal Health (Dublin, Ireland). Solid CEQ was purchased from Qilu Animal Health Products (Jinan City, China). Acetonitrile (CAN, HPLC grade) was purchased from Fisher Scientific (Pittsburgh, PA). Purified water was produced using a MilliQ gradient® Plus Millipore system.

### Animals

Wistar mice of either sex, weighing (160–180 g) were obtained from Daren Fucheng (Qingdao, China). The mice were maintained under standard environmental conditions at 24 ± 2 °C with free access to food and water according to the Guidelines of Experimental Animal Care issued by the Animal Welfare and Research Ethics Committee at Qingdao Agriculture University.

### Microsphere preparation

CEQ-PLGA-MS (microspheres) were prepared by atomization and spray drying as described previously with slight modifications (Beck-Broichsitter et al., 2012). Briefly, microspheres were produced from a solid-in-oil dispersion using a Lab Spray-Dryer SY-6000 (Shang Hai Shiyuan Bio-Engineering, China), PLGA (1.2 g) and 20 ml of dichloromethane were combined under continuous stirring and solid CEQ (0.24 g) was then directly dispersed into the PLGA solution yielding a 6% (w/v) PLGA solution. The polymer/drug solution was mixed using an ultrasonic cell disruptor (Model JY92-II) at 600 W for 10 cycles of 2 s on and 4 s off. The dispersion was spray-dried in a Lab Spray-Dryer SY-6000 (Shang Hai, China) using an inlet air temperature of 40 °C, drying air flow rate of 700 NL/h and an injection rate of 4 ml/min. The equipment was stabilized for 30 min before spray drying. A cyclone separator was used to collect the spray-dried product. Particles were collected only from the collection jar and the bottom 5-cm portion of the cyclone. The resulting microspheres were packaged in ampoules and desiccated for storage.

### Analysis of appearance and size distribution of CEQ-PLGA-MS

The morphology of the prepared CEQ microspheres was examined using a JEOL Model 7500 F Field Emission Scanning Electronic Microscope (SEM) (Tokyo, Japan). Briefly, microspheres were fixed on a brass cylindrical platform using double-sided adhesive tape, and made electrically conductive by vacuum coating a thin gold layer (∼2–4 nm) for 30 s at 40 W. The images of microsphere surfaces or cross-sections were taken at an excitation voltage of 4 kV (Desai & Schwendeman, 2013). Five hundred particles were measured for particle size distribution and measurements were carried out using SEM.

### CEQ-loading and encapsulation efficiencies

One-hundred milligrams of the prepared microspheres were transferred to a 50 ml volumetric flask for the preparations. PLGA microspheres were dispersed in 1 mL dichloromethane and vortexed for 2 min. Deionized H_2_O (25 mL) was added to the solution and then ultrasonicated at 800 W, working 4 s on, 4 s off for 50 cycles. The resulting dispersion was maintained by horizontal shaking at 100 RPM for 1 h at room temperature to fully extract CEQ. The dispersion was diluted to 50 ml with deionized H_2_O and then filtered using a Millex HV 0.22 μm PVDF syringe filter (Millipore, Billerica, MA). The CEQ concentration was analyzed by high-performance liquid chromatography (HPLC).

The analyses of samples using an Agilent Technologies HPLC system (series 1260) with a UV absorbance detector (Agilent Technologies, Santa Clara, CA) set at 269 nm. The mobile phase was sodium perchlorate-H_3_PO_4_-triethylamine (pH 3.6):acetonitrile (85:15 v/v). An Inert Sustain C18 stable bond column (4.6 × 250 mm, 5 μm) (Agilent Technologies) was used with a flow rate of 1.0 mL/min. An injection volume of 20 μL was used for drug-loaded samples. Chromatographs were analyzed using Open Lab CDS ChemStation (Agilent Technologies). CEQ loading was expressed as mg drug/100 mg of CEQ-PLGA-MS (w/w). All experiments were conducted in triplicate.

CEQ entrapment efficiency (%) was calculated as follows: CEQ loading ratio × quantity of CEQ -loaded microspheres/CEQ quantity added in the microsphere preparation process.

### Stability of CEQ-PLGA-MS

Microsphere powders were kept for 6 months at 3–5 °C and 20–30 °C. The microsphere morphology and drug content were tested at 0, 2, 4, and 6 months.

### *In vitro* drug release

The *in vitro* release of CEQ from the microspheres was carried out from both ends of the closed dialysis system. CEQ-loaded PLGA microspheres (50 mg) were added into a dialysis bag (MW cutoff 8–14 kDa; Millipore) and dispersed into 2 mL of PBS (pH 7.4). The sealed dialysis bag was immersed fully in 250 mL of PBS (pH 7.4). The system was agitated at 37 ± 1 °C in a shaking water bath set at 100 rpm for 48 h in the dark. At predetermined time intervals (0.083, 0.25, 0.5, 1, 2, 4, 8, 12, 24, 36 and 48 h), medium was withdrawn in 2 mL volumes and replaced with an equal volume of fresh release medium. The samples were filtered and the CEQ content were determined by HPLC as described above. All release experiments were performed in triplicate and the results are presented as the mean ± standard deviation (SD). The results of all measurements were used to calculate cumulative drug release.

In order to understand the mechanism of CEQ from PLGA microspheres, the *in vitro* drug release data was analyzed by the four kinetic models: zero order (Q_t_=k_0_t), first order [Ln(1 − Q_t)_= −k_1_t], Higuchi (Q_t_=k_H_t^1/2^) and Korsmeyer–Peppas (lnQ_t_ =nlnt + k) (Korsmeyer et al., 1983).Where Q_t_ is the cumulative percentage of drug release at time t, k is the release rate constant, n is the release exponent indicate that mechanism of drug release. Regression analysis was performed and the coefficient of determination (*R*^2^) was calculated to evaluate the kinetic models. The model which showed the highest value of *R*^2^ was regarded to be the most suitable kinetic model for explaining the mechanism of CEQ released from the PLGA microspheres.

### Tissue distribution of microsphere

All animal studies were conducted according to the experimental practices and standards approved by the Animal Welfare and Research Ethics Committee at Qingdao Agriculture University.

For the tissue distribution study, mice were divided into three groups. The blank control group was injected intravenously (i.v.) with sterile PBS (pH 7.4), the drug control group was given CEQ 2.5% solution i.v., and the test group the CEQ-PLGA-MS suspension dispersed in sterile PBS as well as blank PLGA microsphere suspensions. Each formulation was given intravenously at a dose of 12 mg/kg CEQ. Mice were sacrificed in a CO_2_ chamber at 0.083, 0.25, 0.5, 1, 2, 4, 6, 8, 10, 12, 14, 16, 20, 24, 36 and 48 h after dosing. Tissues and organs (heart, liver, spleen, lung, and kidney) were rinsed with PBS and dried with tissue paper to remove excess fluid. Residual fat was also removed and tissue samples were stored at −80 °C until analysis.

### Tissue sample analysis

Mouse tissues (heart, liver, spleen, lung, and kidney) were thawed at room temperature. Ten milligram tissue and 1 ml of PBS (pH 7.4) were combined and homogenized (Silverson SL2 homogeniser) at 10 000 rpm for 1 min. About 0.5 g homogenate was combined with 1 mL 95% acetonitrile in H_2_O (v/v) in a 1.5 mL polypropylene (pp) centrifuge tube and then vortexed for 1 min. After centrifugation at 12 000 rpm for 10 min, extracted three times, supernatants from five tissue samples were combined with 2 mL of hexane and centrifuged as above in pp tubes. The hexane layer was removed and the residual liquid, including a final H_2_O rinse, was loaded onto a C-18 reverse-phase solid phase extraction cartridge (Agela Technologies, Tianjin, China) that had been preconditioned with 2 mL methanol and 2 mL water. The cartridge was washed with 3 mL of acetonitrile/purified water (5:95) and dried under vacuum for 1 min. The analytes were eluted with 2 mL of acetonitrile/H_2_O (15:85). The collected eluate was evaporated to near dryness at 40 °C under nitrogen gas using a Termovap sample concentrator (Hangzhou Allsheng Instruments, Hangzhou, China). The residue was reconstituted in 1 mL H_2_O and filtered (see above) before injection into the UPLC/MS/MS system.

An Agilent 1290 Series HPLC system (Agilent Technologies) equipped with a Zorbax RRHD Eclipse Plus C18 column (2.1 × 50 mm, 1.8 μm) at 30 °C was used for analyte separation. The mobile phase was acetonitrile and 0.1% formic acid in H_2_O (85:15, v/v) with a flow rate of 0.2 mL min^−1^.

The G6460 triple quadruple mass spectrometer (Agilent Technologies) was operated in the positive electrospray ionization mode (ESI+) at 4000 V ion spray voltage. The instrument was tuned and optimized for the transmission of the protonated molecular ions of CEQ (*m*/*z* 529.3) and typical product ions at *m*/*z* 134.1 were obtained. The limit of quantification of CEQ was 3 ng/g, the extraction recoveries of CEQ from tissue samples were >85%, and coefficients of variation were <15% for both within runs and between runs.

### Histopathology

Twelve and forty-eight hours after intravenous administration of CEQ-PLGA-MS to Wistar mice at a dose of 12 mg/kg CEQ, left lungs were collected by dissection and fixed in 4% paraformaldehyde, embedded in paraffin, and cut into 3 μm sections. Sections were stained with hematoxylin and eosin stain (H&E) (McKay et al., 2004). The pathological changes were identified with the aid of the Vectra3.0 Automated Quantitative Pathology Imaging System (Perkin Elmer, Rodgau, Germany).

### Statistical analysis

Values were expressed as mean ± SD for each group. All analyses were performed using SPSS version 12.0 software package (Chicago, IL). Differences were considered to be statistically significant at *p* < 0.05.

## Results and discussion

### The characteristics of CEQ-PLGA-MS

Many drugs are unsuitable for *in vivo* use simply due to their short half-lives and toxicity. Particulate delivery systems such as liposomes and polymeric nano- and micro-particles can alleviate these effects and give sustained delivery of therapeutic agents to the lungs (Chiabc et al., 2015; Zhang et al., 2015). Biodegradable microspheres have been extensively studied for almost half a century and encapsulation using PLGA particles is a successful practice in both clinical and research settings (De Clercq et al., 2016).

There are several methods for generating PLGA particles including spray drying and phase separation/solvent extraction (Aubert-Pouëssel & Venier-Julienne, 2004; Acharya et al., 2009). The latter method is most often used but results in single bulk batches requiring large amounts of reagents, time and labor that results in only a small-batch product. The spray drying process overcomes this limitation.

**Figure 1.**
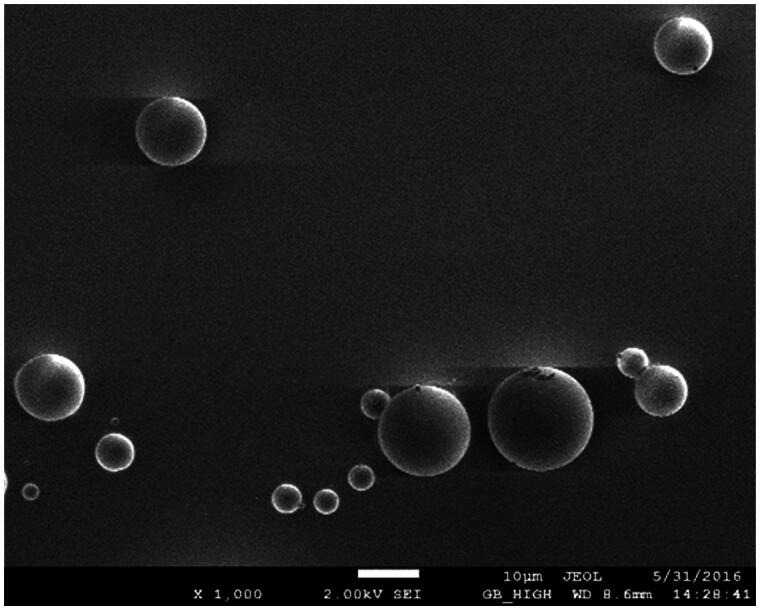
CEQ-loaded microspheres observed by scanning electron microscopy.

PLGA microspheres produced by spray drying have different surface characteristics and drug-loading efficiencies based on material concentrations, the inlet air temperature and the ventilation rate. We identified the optimal formulation and process parameters using an orthogonal optimization design. The optimal formulation was 6% (w/v PLGA)with an inlet air temperature 40 °C, a ventilation rate of 700 NL/h and an injection rate of 4 ml/min. Microspheres prepared using the optimal experimental conditions were globular in appearance and dispersed well (Figure 1). The average drug-loading and encapsulation efficiencies were 18.3 ± 1.32% and 91.6 ± 2.61%, respectively.

### Morphology and drug release

The primary factors influencing microspheres for lung targeting are the particle size and surface characteristics. After intravenous injection, grain sizes of 7 –30 μm are mechanically intercepted by capillary beds and accumulate in the lungs thereby achieving passive lung-targeting (Wang et al., 2014). Our CEQ-PLGA-MS prepared by the spry drying method were discrete and spherical with smooth surfaces (Figure 1). The average particle size was 12.4 ± 1.23 μm, and 87.5% of the microspheres were within the size range of 7–30 μm. These results indicated that the microspheres were suitable for lung accumulation after intravenous injection.

Microsphere storage at 3–5 °C or at room temperature (20–30 °C) for 6 months gave no notable changes either in surface morphology or in drug content.

**Figure 2.**
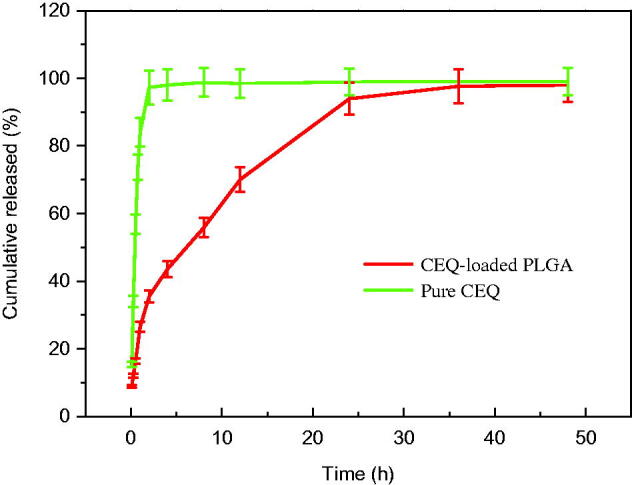
Cumulative CEQ release from CEQ-PLGA-MS in PBS (pH 7.4). *In vitro* release kinetics were obtained at 37 ± 1 °C by dialysis. CEQ release from stock solution was used as control. CEQ loading was 18.3 ± 1.3%. Data as mean ± SD, *n* = 3.

When we examined in *vitro* CEQ release profiles, the microspheres showed three primary discharge processes. The first was an initial burst of CEQ from the sphere surface (up to 1 h). The second was a constant release stage (1–36 h) and the third was no drug release from the microspheres (36–48 h) (Figure 2). Of the total CEQ in the CEQ-loaded microspheres, 24.2% was released during the first hour. This reflected CEQ adsorbed on, or incorporated near, the microsphere surfaces. In clinical practice, this process would result in rapid treatment. After this time, CEQ release was dependent on drug diffusion and polymer degradation.

Our microspheres had a relatively low porosity and were degraded slower which thereby extended the release plateau (Freiberg & Zhu, 2004). After 36 h, almost all CEQ was released and this process was much slower than non-incorporated CEQ in solution (Figure 2). Therefore, the *in vitro* performance of the microspheres showed prolonged and sustained CEQ release.

The mechanism of CEQ release from the PLGA microspheres was investigated by fitting the *in vitro* release data into zero order, first order, Higuchi and Korsmeyer–Peppas models. The coefficient of determination (*R*^2^), release rate constant (k) and n values obtained after regression analysis on four kinetic models are shown in Table 1. It was found that the result was supported by the Korsmeyer–Peppas model as it presented the highest value of *R*^2^ (0.9921). Moreover, the value of n was 0.5663, indicted that a non-Fickian diffusion kinetics (0.5 < *n* < 1) (Peppas, 1985). Thereafter, it is concluded that the drug release mechanism was mainly due to the combination of diffusion of drug through the polymer and polymer degradation of the PLGA microspheres (Erdemli Usanmaz et al., 2014).

**Table 1.** The kinetic models simulated for the release behavior of CEQ-loaded PLGA microspheres.

Model	Equation	*R* ^2^
Zero-order model	*y* = 2.6238*x* + 33.567	0.9789
First-order model	*y* = 0.0285*x* + 3.715	0.8410
Higuchi model	*y* = 14.575*x* + 10.995	0.9543
Korsmeyer–Peppas model	*y* = 0.3564*x* + 1.4359	0.9921

### *In vivo* CEQ distribution

**Figure 3.**
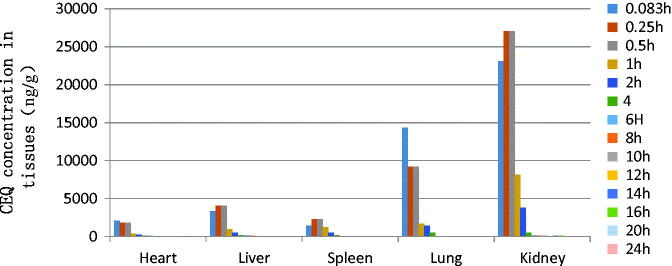
Distribution of CEQ in mouse tissues following i.v. administration of a single dose (6 mg/kg) of CEQ. Each point represents the mean ± SD from six mice.

**Figure 4.**
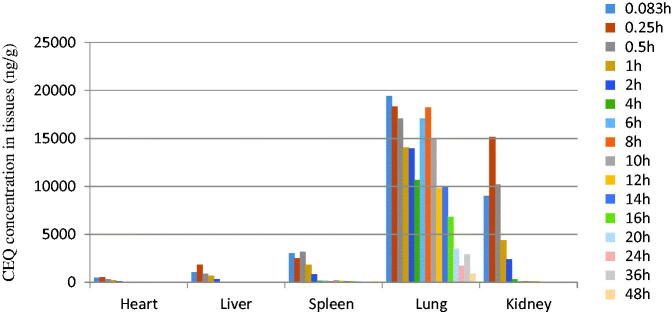
CEQ distribution in mouse tissues following i.v. administration of a single 6 mg/kg dose of CEQ-loaded microspheres. Each point represents the mean ± SD from six mice.

The *in vivo* bio-distribution behavior of CEQ after intravenous injection of the CEQ-loaded microspheres in mice was investigated using CEQ injection as a control. We then determined drug concentrations in the tissues (heart, liver, spleen, lungs and kidney) at various times post-injection. The concentration of CEQ in the kidney was higher than in other tissues in the CEQ solution controls (Figure 3). The total amount of drug accumulated in each organ within 48 h AUC0–48 h of the microspheres was higher than CEQ solution controls in heart, liver, spleen and kidney. In contrast, the CEQ-PLGA-MS delivered CEQ primarily to the lung after intravenous injection (Figure 4).

The main factor influencing microsphere targeting to the lung is the particle size and surface morphology. We found that that our microspheres had an average grain diameter of 12.4 ± 1.23 μm, which was appropriate for lung targeting. This would increase the effective drug levels in the lungs and reduce drug exposure to other tissues. This is propitious for pneumonia treatment and simultaneously reduces side effects.

Based on the above observations, we successfully prepared CEQ-PLGA-MS by the spray drier, which showed both proper lung-targeting and sustained drug release characteristics. Delivery system of CEQ-PLGA-MS is a more viable option than that of nanoparticles, which can bypass the first pass effect, avoid pre-systemic elimination in the liver and epithelial cell toxicity (Luo et al., 2015; Wang et al., 2016). There are many reports that focus on the preparation of lung-targeted microspheres loaded with antibiotics, antitumor drugs, or protein drugs for *in vivo* studies (Lu et al., 2003; Bae et al., 2010; Chifiriuc & Grumezescu, 2012). However, the few studies of lung targeting did not examine many time points after intravenous injection. In our study, tissues were collected at 16 time points after administration giving a more accurate reflection of CEQ-PLGA-MS distribution.

### Histopathological studies

**Figure 5.**
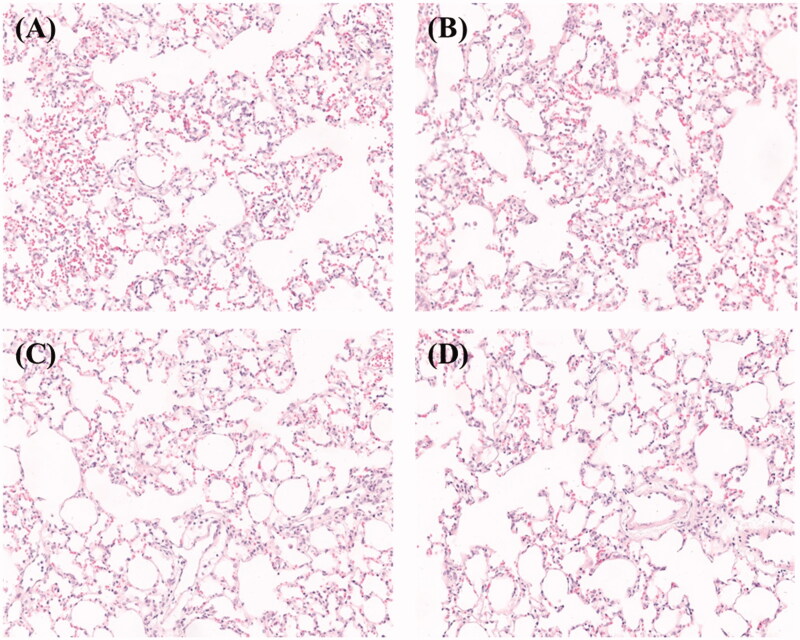
H&E staining of lung tissues i.v. administration of (A,C) CEQ-loaded microspheres or (B,D) microspheres only after (A,B) 12 h and (C,D) 48 h.

To evaluate the impact of microspheres on lung tissue, we performed histopathologic analysis of lungs from mice that received 6 mg/kg dose or blank microspheres as a control. We found that inflammatory cells accumulated in alveolar spaces after 12 h (Figure 5(A), dark blue or purple spots). The inflammation also appeared in lung tissues of mice treated with blank PLGA microspheres (Figure 5(B)).This may indicate the simultaneous embolism of a number of blood vessels by the microspheres (Chen et al., 2014). However, the inflammation disappeared after 48 h (Figure 5(C,D)). In addition, the lung injury disappeared spontaneously as the microspheres decayed.

## Conclusions

We prepared CEQ-loaded microspheres using a spry-drying method that had physiochemical properties and sizes suitable for *in vivo* use. The microspheres showed a combination of lung-targeting and sustained drug release characteristics. The maximum amount of drug was released in the target tissue and caused lung injury that disappeared spontaneously. This work adds to the already significant domain of targeted drug delivery systems that holds a promising alternative over conventional methods of drug delivery.
